# Analysis of muscle magnetic resonance imaging of a large cohort of patient with VCP-mediated disease reveals characteristic features useful for diagnosis

**DOI:** 10.1007/s00415-023-11862-4

**Published:** 2023-08-21

**Authors:** Diana Esteller, Marianela Schiava, José Verdú-Díaz, Rocío-Nur Villar-Quiles, Boris Dibowski, Nadia Venturelli, Pascal Laforet, Jorge Alonso-Pérez, Montse Olive, Cristina Domínguez-González, Carmen Paradas, Beatriz Vélez, Anna Kostera-Pruszczyk, Biruta Kierdaszuk, Carmelo Rodolico, Kristl Claeys, Endre Pál, Edoardo Malfatti, Sarah Souvannanorath, Alicia Alonso-Jiménez, Willem de Ridder, Eline De Smet, George Papadimas, Constantinos Papadopoulos, Sofia Xirou, Sushan Luo, Nuria Muelas, Juan J. Vilchez, Alba Ramos-Fransi, Mauro Monforte, Giorgio Tasca, Bjarne Udd, Johanna Palmio, Srtuhi Sri, Sabine Krause, Benedikt Schoser, Roberto Fernández-Torrón, Adolfo López de Munain, Elena Pegoraro, Maria Elena Farrugia, Mathias Vorgerd, Georgious Manousakis, Jean Baptiste Chanson, Aleksandra Nadaj-Pakleza, Hakan Cetin, Umesh Badrising, Jodi Warman-Chardon, Jorge Bevilacqua, Nicholas Earle, Mario Campero, Jorge Díaz, Chiseko Ikenaga, Thomas E. Lloyd, Ichizo Nishino, Yukako Nishimori, Yoshihiko Saito, Yasushi Oya, Yoshiaki Takahashi, Atsuko Nishikawa, Ryo Sasaki, Chiara Marini-Bettolo, Michela Guglieri, Volker Straub, Tanya Stojkovic, Robert Y. Carlier, Jordi Díaz-Manera

**Affiliations:** 1 Neurology Department, Hospital Clinic de Barcelona, Universitat de Barcelona, Barcelona, Spain; 2 John Walton Muscular Dystrophy Research Centre, Newcastle University Translational and Clinical Research Institute and Newcastle Hospitals NHS Foundation Trust, Center for Life, Central Parkway, Newcastle Upon Tyne, NE13BZ United Kingdom; 3 APHP, Centre de Référence des Maladies Neuromusculaires, Institut de Myologie, Centre de Recherche en Myologie, Sorbonne Université, APHP, Hôpital Pitié-Salpêtrière, Paris, France; 4 Department of Radiology, Assistance Publique-Hôpitaux de Paris (AP-HP), DMU Start Imaging, Raymond Poincaré Teaching Hospital, Garches, France; 5 Département de Neurologie Hôpital Raymond-Poincaré Garches France Inserm U1179, Garches, France; 6 Servicio de Neurología. Hospital Virgen de la Candelaria, Tenerife, Spain; 7 Neuromuscular Diseases Unit, Neurology Department, Institut d’Investigació Biomèdica Sant Pau (IIB SANT PAU), Hospital de la Santa Creu i Sant Pau, Barcelona, Spain; 8 Center for Biomedical Network Research on Rare Diseases (CIBERER), Instituto de Salud Carlos III, Madrid, Spain; 9 Unidad de Enfermedades Neuromusculares, Servicio de Neurología, Instituto de Investigación imas12, Hospital 12 de Octubre, Madrid, Spain; 10 Unidad de Enfermedades Neuromusculares, Servicio de Neurología, Hospital Virgen del Rocio, Seville, Spain; 11 Centro de Investigación Biomédica en Red en Enfermedades Neurodegenerativas (CIBERNED), Barcelona, Spain; 12 Department of Neurology, Medical University of Warsaw, ERN EURO NMD, Warsaw, Poland; 13 UOC di Neurologia e Malattie Neuromuscolari, AOU Policlinico “G. Martino”, Rome, Italy; 14 Neurologie, Neuromusculair Referentiecentrum, Universitaire Ziekenhuizen, Leuven, Belgium; 15 Neurology Department, University of Pécs, Pécs, Hungary; 16 Université Paris Est, U955 INSERM, Centre de Référence de Pathologie Neuromusculaire Nord-Est-Ile-de-France, Henri Mondor Hospital, EURO-NMD, 94010 Creteil, France; 17 Neurology Department, Universitary Hospital Antwerpen, Edegem, Belgium; 18 Department of Neurology, Eginition Hospital, Medical School, NKUA, ERN, EURO NMD, Athens, Greece; 19 Neurology Department, Huashan Hospital, Fudan University, Shangai, China; 20 Neuromuscular Diseases Unit, Neurology Department, Hospital Universitari I Politècnic La Fe, Valencia, Spain; 21 Neuromuscular Reference Centre, ERN-EURO-NMD, Warsaw, Poland; 22 Neuromuscular and Ataxias Research Group, Instituto de Investigación Sanitaria La Fe, Valencia, Spain; 23 Department of Medicine, Universitat de València, Valencia, Spain; 24 Unitat de Malalties Neuromusculars, Servei de Neurologia, Hospital Germans Tries I Pujol, Badalona, Spain; 25 UOC di Neurologia, Fondazione Policlinico Universitario A. Gemelli IRCCS, Rome, Italy; 26 Tampere Neuromuscular Center, Tampere University Hospital, Tampere, Finland; 27 Folkhalsan Genetic Institute, Helsinki University, Helsinki, Finland; 28 Sree Chitra Tirunal Insitute for Medical Sciences and Technology, Thiruvananthapuram, India; 29 Department of Neurology, Friedrich-Baur-Institute, LMU Clinics, Munich, Germany; 30 Neurology Department, Biodonostia Health Research Institute, Donostia, Spain; 31 Department of Neurosciences, University of Padova, Padua, Italy; 32 Department of Neurology, Institute of Neurological Sciences, Queen Elizabeth University Hospital, Glasgow, Scotland, UK; 33 Heimer Institut for Muscle Research, Klinikum Bergmannsheil Ruhr, University Bochum, Bochum, Germany; 34 University of Minnesota, Minneapolis, USA; 35 Centre de Référence des Maladies Neuromusculaires Nord/Est/Ile-de-France and ERN-EURO-NMD, Neurology Department, Hôpitaux Universitaires de Strasbourg, Strasbourg, France; 36 Neurology Department, Medical University of Vienna, Vienna, Austria; 37 Leiden University Medical Center, Leiden, The Netherlands; 38 Department of Medicine (Neurology), The Ottawa Hospital, Ottawa, Canada; 39 Departamento de Neurología y Neurocirugía, Hospital Clínico Universidad de Chile, Santiago de Chile, Chile; 40 Department of Neurology, Johns Hopkins University School of Medicine, Baltimore, USA; 41 Department of Neuromuscular Research, National Institute of Neuroscience, National Center of Neurology, Tokyo, Japan; 42 Department of Neurology, National Center Hospital, NCNP, Tokyo, Japan; 43 Department of Neurology, Kagawa Prefectural Central Hospital, Kagawa, Japan; 44 Department of Neurology, Kinki Central Hospital, Hyogo, Japan; 45 Department of Neurology, Graduate School of Medicine, Dentistry and Pharmaceutical Sciences, Okayama University, Okayama, Japan

**Keywords:** VCP myopathy, Multisystemic proteinopathy, Muscle MRI, Valosin

## Abstract

**Background:**

The diagnosis of patients with mutations in the *VCP* gene can be complicated due to their broad phenotypic spectrum including myopathy, motor neuron disease and peripheral neuropathy. Muscle MRI guides the diagnosis in neuromuscular diseases (NMDs); however, comprehensive muscle MRI features for *VCP* patients have not been reported so far.

**Methods:**

We collected muscle MRIs of 80 of the 255 patients who participated in the “VCP International Study” and reviewed the T1-weighted (T1w) and short tau inversion recovery (STIR) sequences. We identified a series of potential diagnostic MRI based characteristics useful for the diagnosis of *VCP* disease and validated them in 1089 MRIs from patients with other genetically confirmed NMDs.

**Results:**

Fat replacement of at least one muscle was identified in all symptomatic patients. The most common finding was the existence of patchy areas of fat replacement. Although there was a wide variability of muscles affected, we observed a common pattern characterized by the involvement of periscapular, paraspinal, gluteal and quadriceps muscles. STIR signal was enhanced in 67% of the patients, either in the muscle itself or in the surrounding fascia. We identified 10 diagnostic characteristics based on the pattern identified that allowed us to distinguish *VCP* disease from other neuromuscular diseases with high accuracy.

**Conclusions:**

Patients with mutations in the *VCP* gene had common features on muscle MRI that are helpful for diagnosis purposes, including the presence of patchy fat replacement and a prominent involvement of the periscapular, paraspinal, abdominal and thigh muscles.

**Supplementary Information:**

The online version contains supplementary material available at 10.1007/s00415-023-11862-4.

## Introduction

The *VCP* gene encodes the valosin-containing protein (VCP), a member of the ATPases Associated with diverse cellular Activities (AAA +) family of proteins. VCP is ubiquitously expressed, and it is involved in protein degradation by the ubiquitin–proteasome system and in cellular homeostasis regulation [1–3]. The group of Dr. Kimonis described in 2001 that mutations in the *VCP* gene were the cause of an autosomal dominant disease characterized by the combination of an inclusion body myopathy (IBM), Paget's disease of the bone (PDB) and frontotemporal dementia (FTD), also known as IBMPFD [4–7]. Since the original description, many other phenotypes and diseases have been reported associated with mutations in the *VCP* gene including facio-scapulo-humeral muscle weakness, distal myopathy, amyotrophic lateral sclerosis, parkinsonism, hereditary spastic paraplegia, Charcot–Marie–Tooth disease type 2, Huntington´s disease and cardiomyopathy [8–17]. Because of this wide range of clinical presentations, the IBMPFD acronym was replaced by the term multisystem proteinopathy (MSP) to encompass all the phenotypes associated with *VCP* mutations [6]. Other genes have recently been described to also cause MSP including *hnRNPA1, SQSTM1*, *MATR3*, *TIA1* and *OPTN* [18, 19]. To better understand the variable features of MSP produced by mutations in the *VCP* gene (*VCP*-MSP), we collected demographic, clinical, genetic, muscle MRI and muscle biopsy data of 255 patients from different countries included in the “VCP International Study” [20]. Clinical and genetic data of the whole cohort have already been described, confirming that the muscles affected at disease onset are very variable among patients and can include the limb girdle, axial or distal muscles. However, muscle weakness quickly spreads with disease progression and can potentially affect almost all muscles at disease stages including respiratory muscles. This phenotypic variability makes the diagnosis challenging, especially if patients present with atypical symptoms without clear family history [21].

Muscle MRI is widely used to evaluate patients with neuromuscular diseases as it can provide helpful information about structural changes including increase in water content which is commonly linked to active muscle fiber necrosis, inflammation or denervation, and fat replacement, which is linked to chronic irreversible loss of muscle tissue [22]. Information obtained from the MRI complements that obtained from muscle function assessments and helps provide tailored care for patients. Moreover, identification of a selected pattern of muscle involvement by muscle MRI can be very useful for diagnostic purposes [23]. MRI sequences quantify structural changes in skeletal muscle of patients with neuromuscular diseases and are gaining popularity as an outcome measure both for clinical trials and natural history studies [24, 25]. However, little is known about the muscle MRI features in patients with *VCP*-MSP disease, as most of the published data is based on single cases or small cohorts [26–29]. Additionally, MRI can also play a role in these patients by identifying changes in the bone characteristic of PDB. There is a large amount of literature describing the radiological features of PDB using conventional X-ray and CT scans. Findings include osseous dedifferentiation, coarsened trabeculae, cortical thickening, bone condensation and enlargement [30]. PDB can have an asymmetric distribution and affect multiple bones most commonly the lumbar spine, pelvis, sacrum, femur and the skull [31]. Bone scintigraphy is also sensitive as it can detect an increased blood flow and associated osteoblastic and osteoclastic activity seen in PDB [32]. Bone MRI is not commonly used for diagnostic purposes in PDB, although it can detect changes compatible with the disease [33]. The major indication for performing an MRI in the follow-up of PDB patients is to detect malignant transformation of a lesion. T1-weighted (T1w) as well as fat-suppressed T2-weighted (T2w) images are useful for this purpose: T1w MR images can show bone enlargement, cortical thickening within maintained yellow marrow and fat-suppressed T2w images display abnormal intermediate and heterogeneous high signal intensity of bone marrow [30]. To our knowledge, there are no published studies using MRI to identify the bone lesions in patients with mutations in the *VCP* gene. Here, we describe the muscle and bone MRI features of a large cohort of patients with mutations in the *VCP* gene that were included in the “VCP International Study” and provide relevant information useful for diagnostic purposes [20].

## Methods

### Study setup and subjects

The “VCP International Study” is a multicenter collaborative retrospective study collecting demographic, genetic, clinical, MRI and muscle biopsy data of patients with a genetically confirmed diagnosis of MSP caused by mutations in *VCP* gene [20].

Inclusion criteria for participating in the “VCP International Study” were: (i) patients having a pathogenic (P)/likely pathogenic (LP) monoallelic variant in the *VCP* gene and (ii) enough data available in the clinical notes about demographic and clinical data as stated in the original description of the cohort [20]. For this MRI study, we selected all patients that had a muscle MRI performed during their follow-up for diagnostic purposes.

### Clinical and genetic data

We reviewed the demographic and clinical data available including gender, age at first symptom, age at MRI, phenotype of the disease, pattern and severity of muscle weakness when the MRI was performed, associated diagnosis of PDB, involvement of the central nervous system (dementia, parkinsonism, upper motor neuron involvement) and diagnosis of polyneuropathy. The specific mutation detected in the *VCP* gene was also collected.

### MRI acquisition and analysis

All patients were scanned in 1.5 or 3 Tesla MRI machines using previously published standardized protocols to acquire T1w and STIR images of the lower limbs or the whole body, although the vendors and coils used varied from one center to another [34]. For scoring purposes, we requested a minimum of 3 axial slices of each muscle covering the maximum volume possible from the proximal to the distal insertion of each muscle. Patients from whom only single MRI slices were provided were excluded as well as MRIs of bad quality where individual muscles could not be clearly identified. All scans were evaluated and independently judged by an experienced neuroradiologist (R. C.) and a neurologist (D. E.), who were blinded to the clinical information using the same protocol of analysis. First, general features of the structural changes on the MRIs were assessed including texture of fat replacement (widespread or patchy), asymmetries in fat replacement (two or more points difference in the score described below between both sides) and existence of a gradient of muscle involvement. Second, muscle fat replacement was scored using a semiquantitative visual score on axial T1w images consisting of 1 to 4 grades as follows: normal muscle appearance (score 1); occasional scattered areas of increased density in < 30% of the muscle volume (score 2); numerous discrete areas of increased density in 30–60% of the muscle volume (score 3); and washed-out appearance due to increased areas of confluent density in more than 60% of the muscle volume (score 4). Inter-rater agreement kappa between the two assessors was 0.75 (95% CI 0.73–0.77, *p < *0.001). In the case of a disagreement in the score, the muscle was reviewed by the two assessors who agreed on a final score. Third, STIR images were reviewed to identify signal hyperintensities that were categorized as absent (score 0), mild (score 1), moderate (score 2) and severe (score 3). Finally, both T1w and STIR images were systematically reviewed to detect changes compatible with PDB by an experienced radiologist (R.C.).

To investigate correlations between the degree of fat replacement and clinical or genetic features we calculated a cumulative MRI score per patient, named here as MRI score, by adding the score of fat replacement for each muscle.

### Modeling the data to identify characteristics for diagnosis

The analysis of fat replacement allowed us to identify groups of muscles that were commonly affected and muscles that were commonly spared, proposing a group of diagnostic characteristics. We assessed the accuracy of these rules in the MRIs obtained from the “VCP International Study” cohort and also in a group of 1089 muscle MRI scans of patients with 10 different neuromuscular diseases including Duchenne/Becker muscular dystrophy, limb girdle muscular dystrophy (LGMD) R1 (*CAPN3*), LGMD-R2 (*DYSF*), LGMD-R3 to 6 (caused by mutations in the sarcoglycan genes), LGMD-R9 (*FKRP*), LGMD-R12 (*ANO5*), facioscapulohumeral muscular dystrophy (FSHD), Pompe disease, oculopharyngeal muscular dystrophy (OPMD) and patients with a myopathy caused by mutations in the *LMNA* gene that were used to build the Myo-Guide artificial intelligence algorithm published in 2020 [35].

We developed an algorithm that integrated these rules into a single predictive model. The algorithm contained a group of rules (*R*) that included all single diagnostic rules (*r*) as follows: *R = *[*r*_0,  *r*_1, … *r*_*n*]. R was applied to each patient (p) obtaining a score of *r = *0 if the patient did not meet the rule or *r = *1 if the patient did meet the rule. A receiver operating characteristic curve (ROC) was used to represent the results of the algorithm applied to our cohort. In order to reduce the impact of the missing data on the results of the algorithm, we applied it, but considering the best- and worst-case scenarios defined as Best-Case Scenario (BCS) when all missing data (NAs) in *VCP*-MSP disease patients were True, while all NAs in other diseases were False, and the Worst-Case Scenario (WCS) when all NAs in positive class (VCP-MSP) were False, while all NAs in negative class were True. A more detailed description of how the algorithm was developed can be found in the supplemental methods section.

### Statistics

We used the Shapiro–Wilk test to study whether the variables were normally distributed. We used parametric studies for variables normally distributed and nonparametric studies for variables non-normally distributed. The specific test used for each analysis is mentioned in the text. If a high number of comparisons were studied, Bonferroni correction was used as post hoc analysis. Hierarchical analysis and graphical representation as a heatmap were performed using R software, V.4.2.1 (https://www.r-project.org). Statistical analyses were performed using IBM SPSS Statistics, V.21 (IBM, Armonk, New York, USA). Quadratic weighted kappa was calculated to assess inter-rater agreement. A *p < *0.05 level of significance was allowed. Statistical analyses were performed using SPSS for Windows version 22.0.

## Results

### Patients

We collected MRI scans from 80 of the 255 patients included in the “VCP International Study.” Nine MRI scans were excluded due to low quality or lack of enough slices to adequately assess muscle involvement leaving a group of 71 MRI scans that were finally included in the study. All patients except for one asymptomatic carrier had some degree of muscular weakness at the time of MRI. Symptomatic patients had a mean onset of symptoms at 45 years (range 18–67) with a mean time from onset of symptoms to the MRI of 7.6 years (range 0–29).

Of the 70 symptomatic patients included in the study, 53 patients had an isolated myopathy, and 17 patients had a myopathy combined with signs of motor neuron involvement of which 12 had exclusive upper motor neuron involvement, five had a combination of upper and lower motor neuron involvement, and two had exclusive involvement of the lower motor neuron. The pattern of muscle weakness at onset was very variable across patients, the most common being generalized weakness affecting upper and lower limbs in 13 cases. The frequency of the mutations of patients included in the study can be found in Supplemental Table 1.

### MRI analysis: general features

Of the 71 MRI scans included in the study, 20 were whole-body MRIs, three included upper and lower limbs but not trunk muscles and 48 included images of the lower limbs only. Signal abnormalities in T1w images were detected in all but one patient (70/71). The normal MRI was of an asymptomatic carrier with no muscular weakness at the age of 46 years old.

**Fig. 1:**
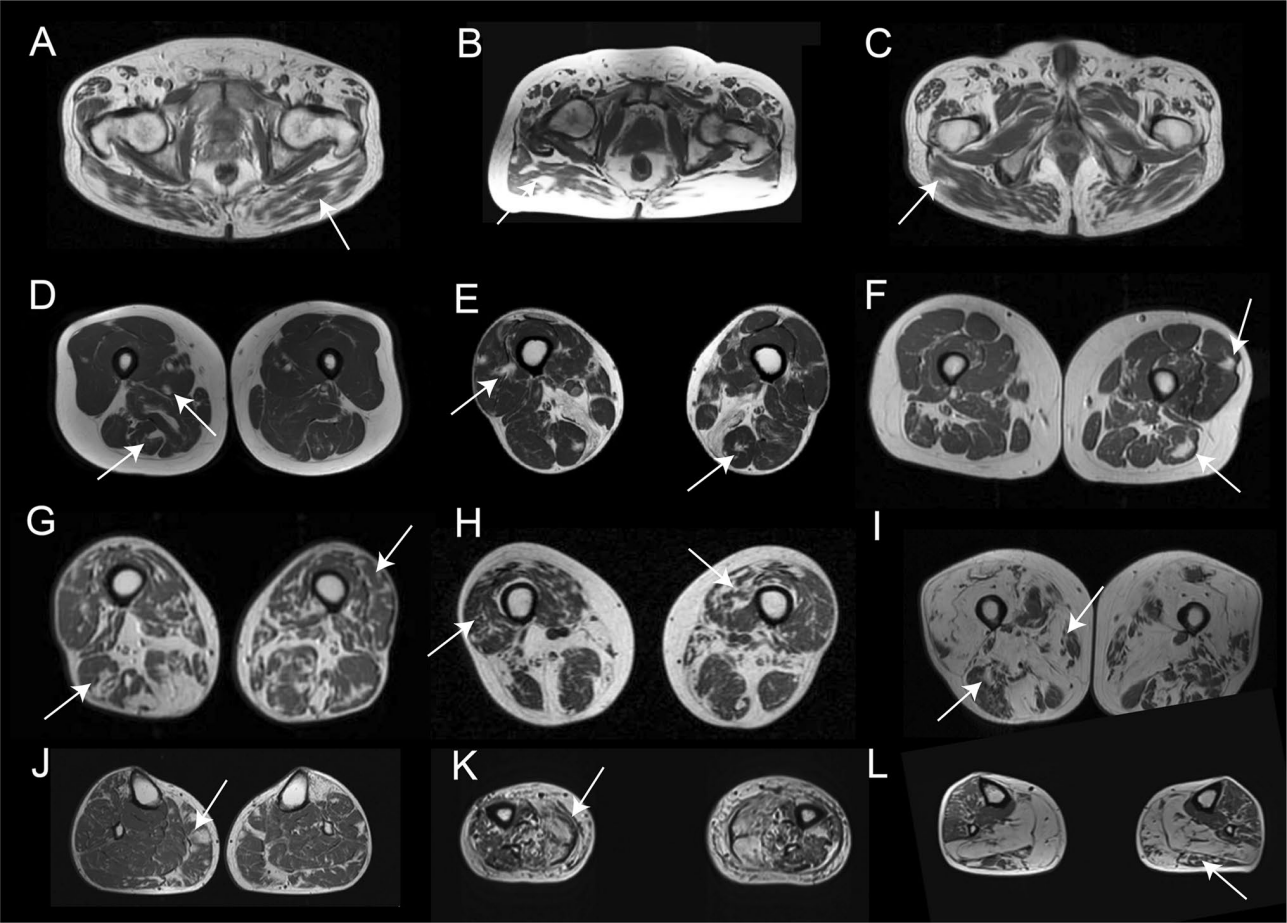
Distribution of fat replacement in the skeletal muscles of patients with mutations in the VCP gene. The figure shows examples of the distribution of fat replacement in patients with mutations in the VCP gene at different stages of disease progression. **A**, **B** and **C** show examples of fat replacement in the *gluteus maximus*. **D**, **E** and **F** show patients at early stages of disease progression where fat can be seen as spots of fat in the muscles as highlighted by the arrows. **G**, **H** and **I** show images of fat replacement in the thigh of patients at middle (**G** and **H**) and advanced (**I**) stages of disease progression. **J**, **K** and **L** show examples of the fat replacement in the muscles of the legs in patients in early (**J**) and middle (**K** and **L**) stages of disease progression

Patients showed a combination of muscles that were completely/almost completely replaced by fat, muscles that were partially affected and muscles that were spared. In the case of partial replacement, we identified a peculiar distribution characterized by patches of fat replacement in the earliest stages that progressed toward areas of apparently normal muscle embedded on large confluent areas of fat tissue, what has been described as “fat pockets” or “popcorn appearance” (Fig. 1). We identified these “fat pockets” in at least one muscle in 67/70 patients (95.7%) of which 36 patients (42.8%) had them in the thigh, affecting mostly the femoral quadriceps muscles (24 patients, 28.5%), and 57 patients (67.8%) had them in the lower legs, predominantly in the *peronei* (39 patients, 46.4%) and *tibialis anterior* muscles (35 patients, 41.6%). Fat pockets were observed throughout disease progression, from patients with mild changes to patients in very advanced stages of the disease. Additionally, we observed another peculiar distribution of the fat that consisted of a linear distribution of the fat in the coronal slices in some muscles, including the *glutei* muscles, the *vasti* or the *tibialis anterior* muscles (Supplemental Fig. 1).

**Fig. 2:**
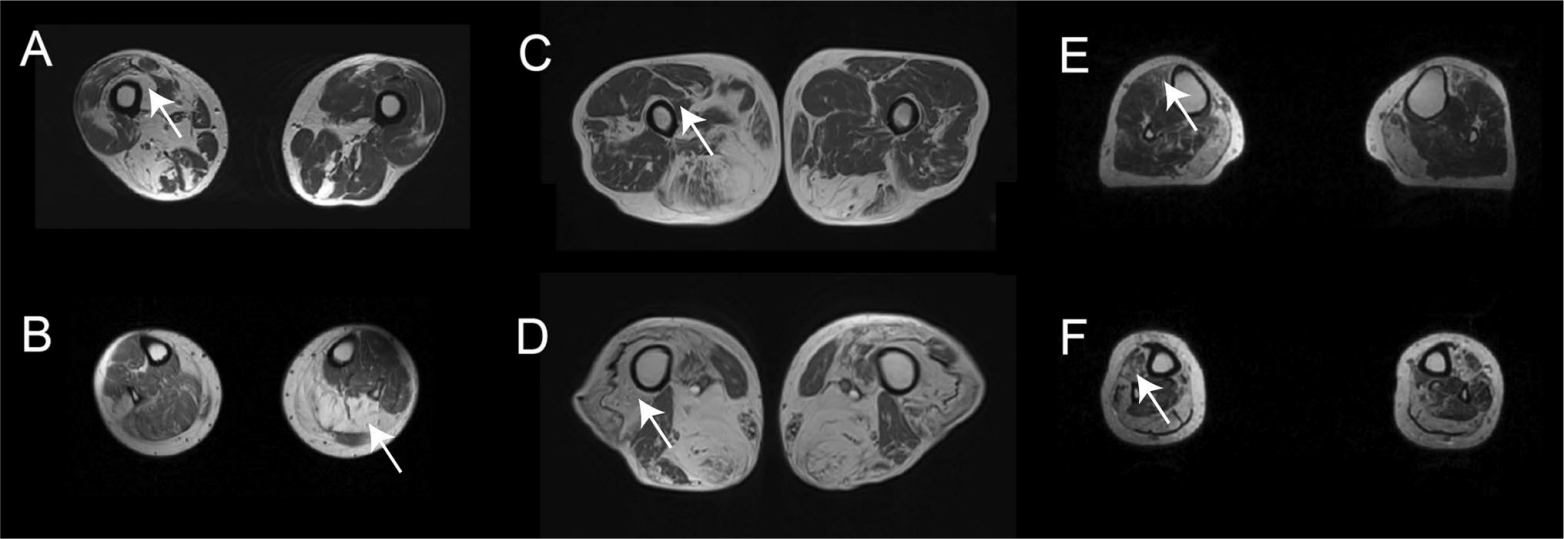
Asymmetric and distal to proximal gradient of fat replacement. **A** and **B** show MRI of two patients with asymmetric fat replacement in the vastus intermedius (arrow in **A**) and the soleus (arrow in **B**) muscles. **C** and **D** show a distal to proximal gradient of fat replacement in the *vastus intermedius* muscle (arrow). **E** and **F** show a distal to proximal gradient of fat replacement in the *tibialis anterior* muscle (arrow)

Asymmetric involvement, judged as a score difference of at least 2 points in at least one muscle, was found in 38 patients (54%), although in 21/38 (55.2%) the asymmetry was found in one muscle only (Fig. 2A and B). Asymmetry in two or more muscles was found in 17/38 patients (44.7%). A distal to proximal gradient of fat replacement was observed in 24 patients (34.2%) and was identified mainly in the *femoral quadriceps* and *soleus* (Fig. 2C–F).

### Pattern of fat replacement: Head, scapula and arm muscles

**Fig. 3:**
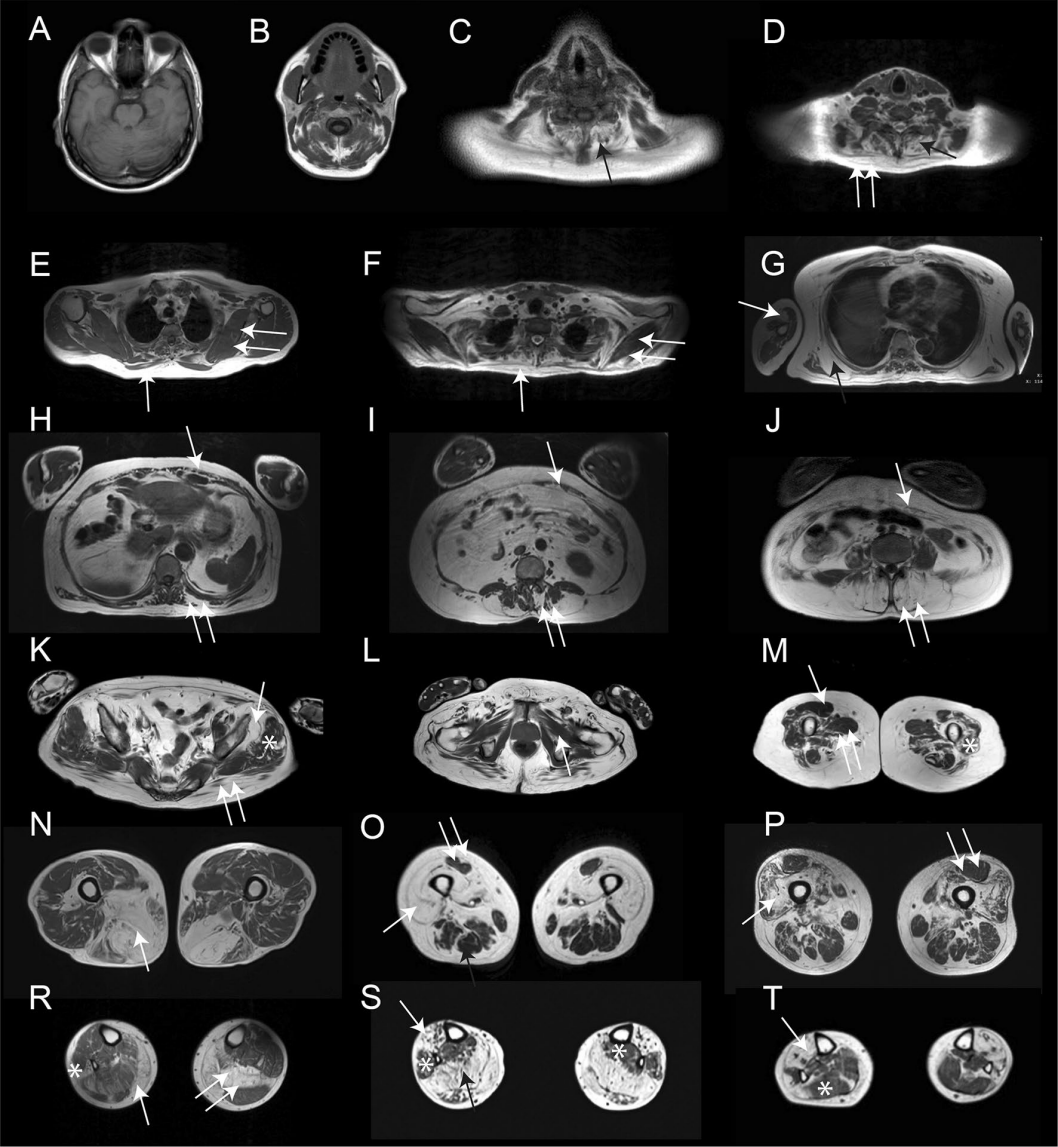
Fat replacement pattern in patients with mutations in the VCP gene. The figure shows the pattern of fat replacement observed in different patients with mutations in the *VCP* gene. **A** and **B** show normal cranial muscles without fat replacement. **C** shows fat replacement of the paraspinal cervical muscles (black arrow). **D** shows fat replacement of the paraspinal cervical muscles (black arrow) and *trapezius* (double arrow). **E** and **F** show a fat spot in the *trapezius* muscle (arrow) while scapular muscles are spared (double arrow). G shows fat replacement of the *serratus anterior* (black arrow) and the *biceps brachii* (double arrow). **H**, **I** and **J** show different examples of fat replacement in the trunk muscles, including abdominal (arrow) and paraspinal (double arrow) muscles. **K** shows involvement of the *gluteus maximus* (double arrow) and *gluteus minimus* (arrow) muscles, while *gluteus medius* (asterisk) is spared. **L** shows no involvement of the pelvic floor muscles (arrow). **M** shows characteristic involvement of upper thigh characterized by sparing of *rectus femoris* (arrow) and *adductor longus* (double arrow), with involvement of the vasti muscles (asterisk). **N**, **O** and **P** show different combinations of muscle fatty replacement observed in the thigh, **N** shows predominant posterior thigh involvement (arrow), while **O** and **P** show predominant anterior involvement (arrow) with sparing of *rectus femoris* (double arrow). **R**, **S** and **T** show different combinations of muscle fatty replacement observed in the leg, **R** shows fat replacement of *gastrocnemius medialis* (arrow) associated with asymmetric involvement of *soleus* (double arrow) and *peroneus* (asterisk). **S** shows involvement of the anterior (arrow) and posterior compartment (black arrow) and sparing of *peroneus* and *tibialis posterior* muscles (asterisk). **T** shows predominant anterior involvement (arrow) with sparing of *soleus* (asterisk)

**Fig. 4:**
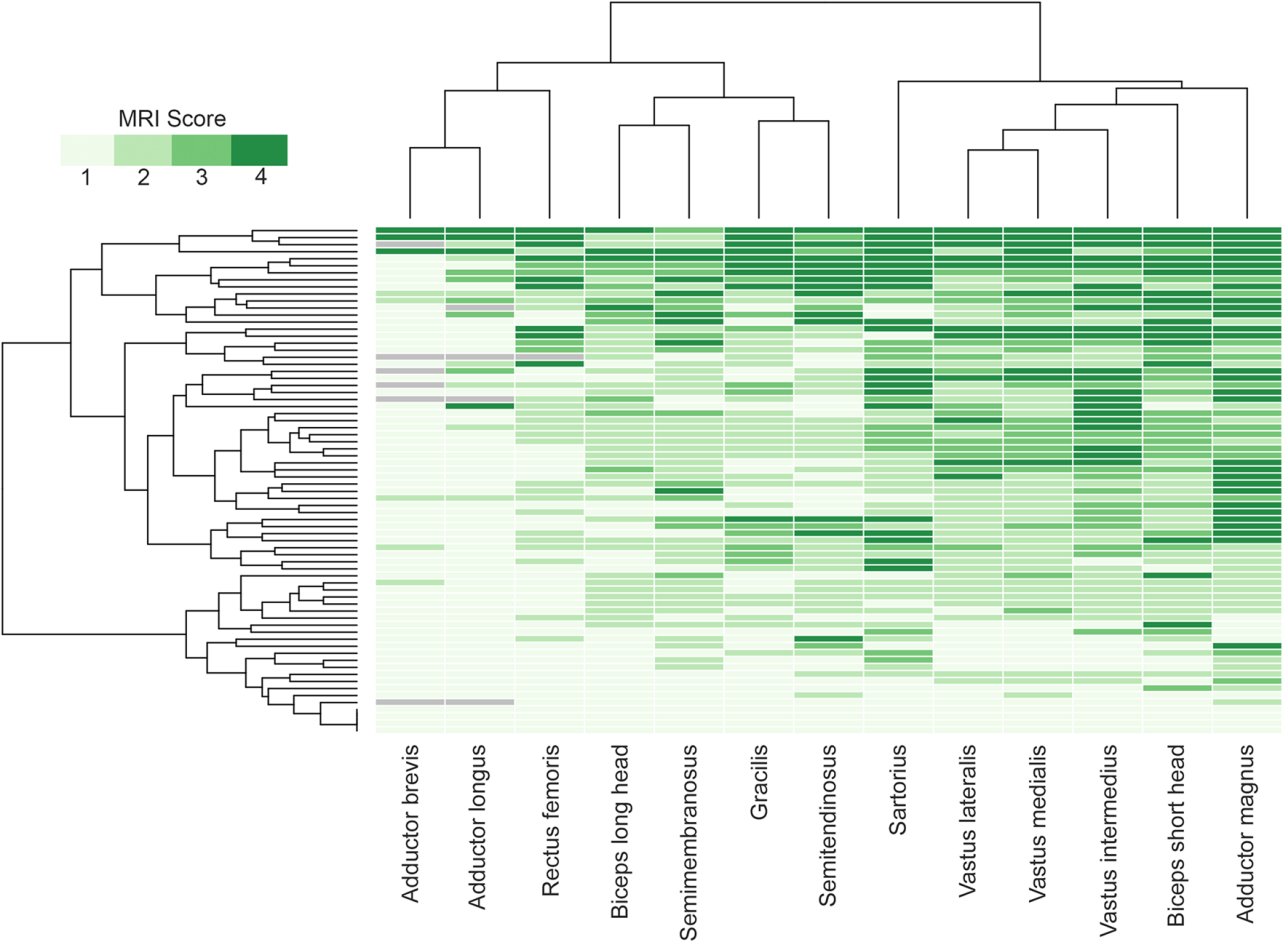
Heatmap showing muscle fatty replacement of muscles of the thigh. Heatmap showing fat replacement of the muscles of the thigh. Patients and muscles are ordered according to hierarchical clustering. The score of a muscle in a patient is indicated by the color of the square. As shown in the figure, adductor magnus, biceps short head, vasti muscles and the sartorius were the most frequently affected muscles, while adductor longus, adductor brevis and rectus femoris were commonly spared or less affected

None of the 20 patients with an MRI available of the head had fat replacement of any facial muscles (Fig. 3). However, fat replacement of the neck extensors was observed in seven of 20 cases. Scapular and arm involvement was analyzed in 23 patients. We identified four muscles to be commonly affected including the *trapezius* in 82.6%, *serratus anterior* in 81.8%, *pectoralis major* in 68.2% and *latissimus dorsi* in 65.2% of the patients (Fig. 4). Interestingly, *supraspinatus, infraspinatus* and *subscapularis* were the most preserved muscles, being involved in 5 to 9% of patients and only to a mild extent. In the arms, the *biceps brachii* and the *deltoid* muscles were the most commonly and severely affected muscles in 45% of the cases (Fig. 3). Distal arm involvement was infrequent. We only found one patient with mild fat replacement of the anterior forearm compartment although this result needs to be taken with caution due to the low number of MRIs that included this region.

### Pattern of fat replacement: trunk and pelvic muscles

Fat replacement in almost one muscle of the trunk was observed in all the 23 patients analyzed. All patients showed involvement of at least one abdominal muscle (Fig. 3). In 94% of the cases there was involvement of paraspinal muscles including *rotator, multifidus, longissimus* and *ilio-costalis* muscles that was defined as fat replacement of score equal or higher than 3 in at least one muscle in one or more spinal segments. The analysis of the whole spinal segments showed a cranio-caudal gradient consisting in the cervical segment being less affected than the thoracic segment and the thoracic segment being less affected than the lumbar segment. This gradient was associated with an incomplete mediolateral gradient of involvement, consisting in the medial part being less affected than the lateral part of the paraspinal musculature as shown in Supplemental Fig. 2.

An MRI covering the whole *psoas, iliacus* and the *glutei* muscles was available for 45 patients. The *Gluteus minimus* was the most frequently and severely affected (85.4%, mean score 2.9) muscle in this region, followed by the *psoas* (62.2%, mean score 1.8) and *gluteus maximus* (53.3%, mean score 1.7). An MRI covering the pelvic floor was available for 57 patients. In general, this region was completely spared or only mildly affected, as only 17 patients (30%) showed fat replacement in one or more muscles and always to a minor extent (mean score 1.3).

### Pattern of fat replacement: thigh muscles

An MRI covering the whole thigh was available for 70 patients. Fat replacement of at least one muscle was observed in 67/70 patients (95.7%). There was a wide variety of muscles affected, but we identified five muscles to be consistently affected in most of the patients including the *adductor magnus*, affected in 62/70 (88.5%, mean score 2.6), the *biceps femoris short head*, affected in 62/70 (88.5%, mean score 2.4), the *vastus intermedius*, affected in 56/70 (80.0%, mean score 2.3), the *vastus medialis*, affected in 57/70 (81.4%, mean score 2.3), and the *vastus lateralis*, affected in 55/70 (78.5%, mean score 2.0) (Fig. 4). In contrast, the *adductor brevis* and *adductor longus* were the least affected muscles, being totally spared in 88% and 80% of patients, respectively. In line with the heterogeneity of clinical involvement, no consistent imaging pattern could be identified in the thigh region. Nevertheless, the combined involvement or sparing of some muscles was frequently observed. For example, the association of the *adductor magnus* together with at least one *vasti* muscle was observed in 91% of the patients, and in 90% of the cases, there was involvement of at least one muscle of the posterior compartment of the thigh, either the *biceps femoris* short head, the *semimembranosus* or the *semitendinosus*. Interestingly, the *sartorius* and *gracilis* muscles were commonly affected, being completely replaced by fat in some patients even though other thigh muscles were still relatively preserved, which could be a useful clue to differentiate *VCP*-MSP disease from other limb girdle muscular dystrophies (LGMD).

### Pattern of fat replacement: leg muscles

**Fig. 5:**
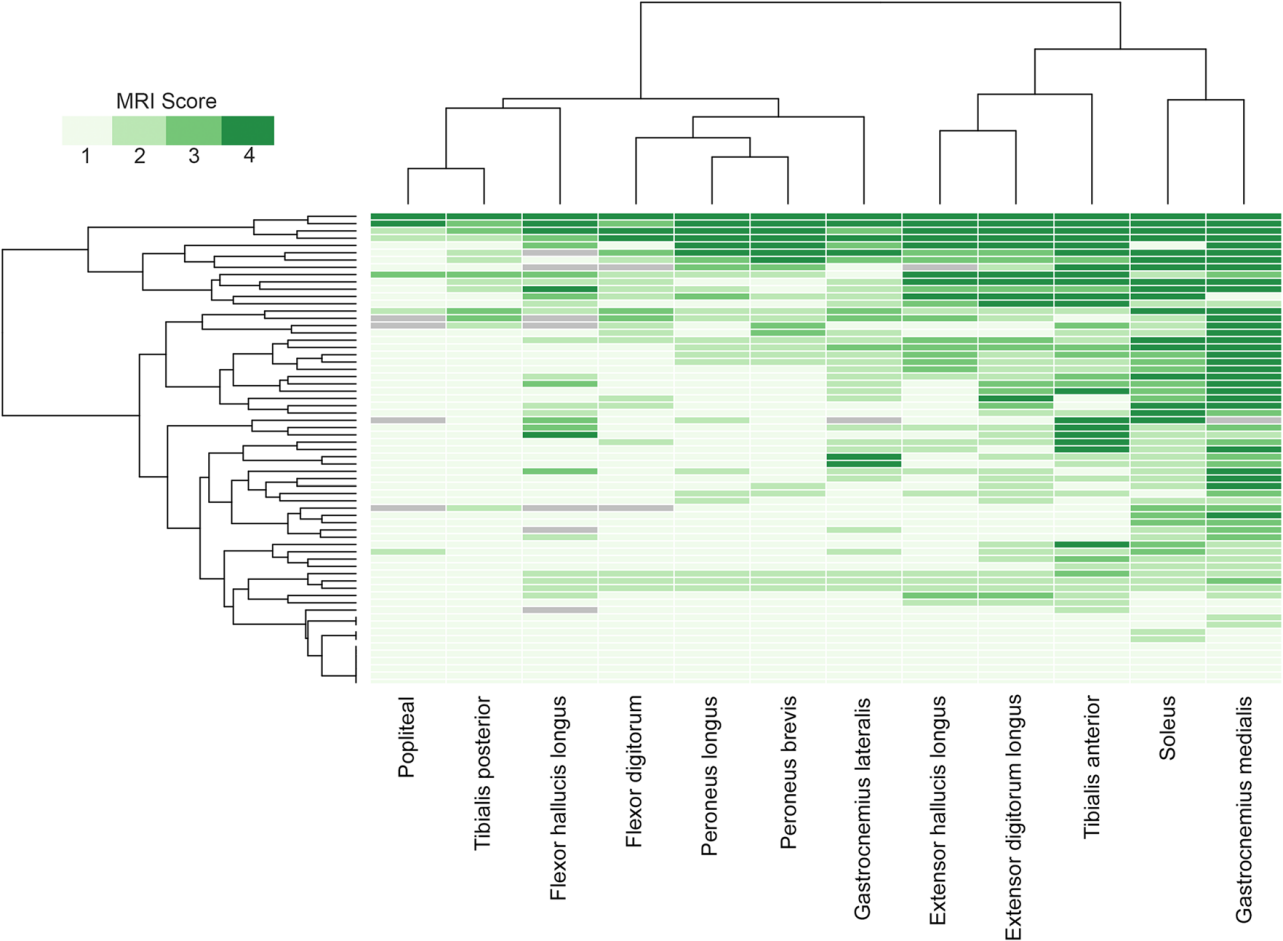
Heatmap showing muscle fatty replacement of muscles of the leg. Heatmap showing fat replacement of the muscles of the leg. Patients and muscles are ordered according to hierarchical clustering. The score of a muscle in a patient is indicated by the color of the square. As shown in the figure, gastrocnemius medialis and soleus were the muscles more commonly affected followed by the distal anterior compartment muscles: tibialis anterior, extensor digitorum longus and extensor hallucis longus, while tibialis posterior and popliteal muscles are commonly spared

An MRI covering the whole leg was available for 60 patients. Fatty replacement of at least one muscle was observed in 54/60 patients (90%). We identified two muscles to be more frequently affected: the *gastrocnemius medialis* muscle was affected in 49/60 (81.6%, mean score 2.5) and the *soleus* in 47/60 (78.3%, mean score 2.2) (Fig. 5). The *tibialis posterior* and the *popliteal* muscles were the least commonly affected muscles in this region*.* As observed in the thigh, there was a wide variability in the muscles affected in the legs from one patient to the other; however, some common combinations were observed. In this sense, involvement of at least one muscle of the calves and one muscle of the anterior compartment was observed in 44/60 patients (73.3%) while isolated involvement of the calves or the anterior compartment was observed in 8/60 and 2/60, respectively (Fig. 3).

### Analysis of STIR sequence

**Fig. 6:**
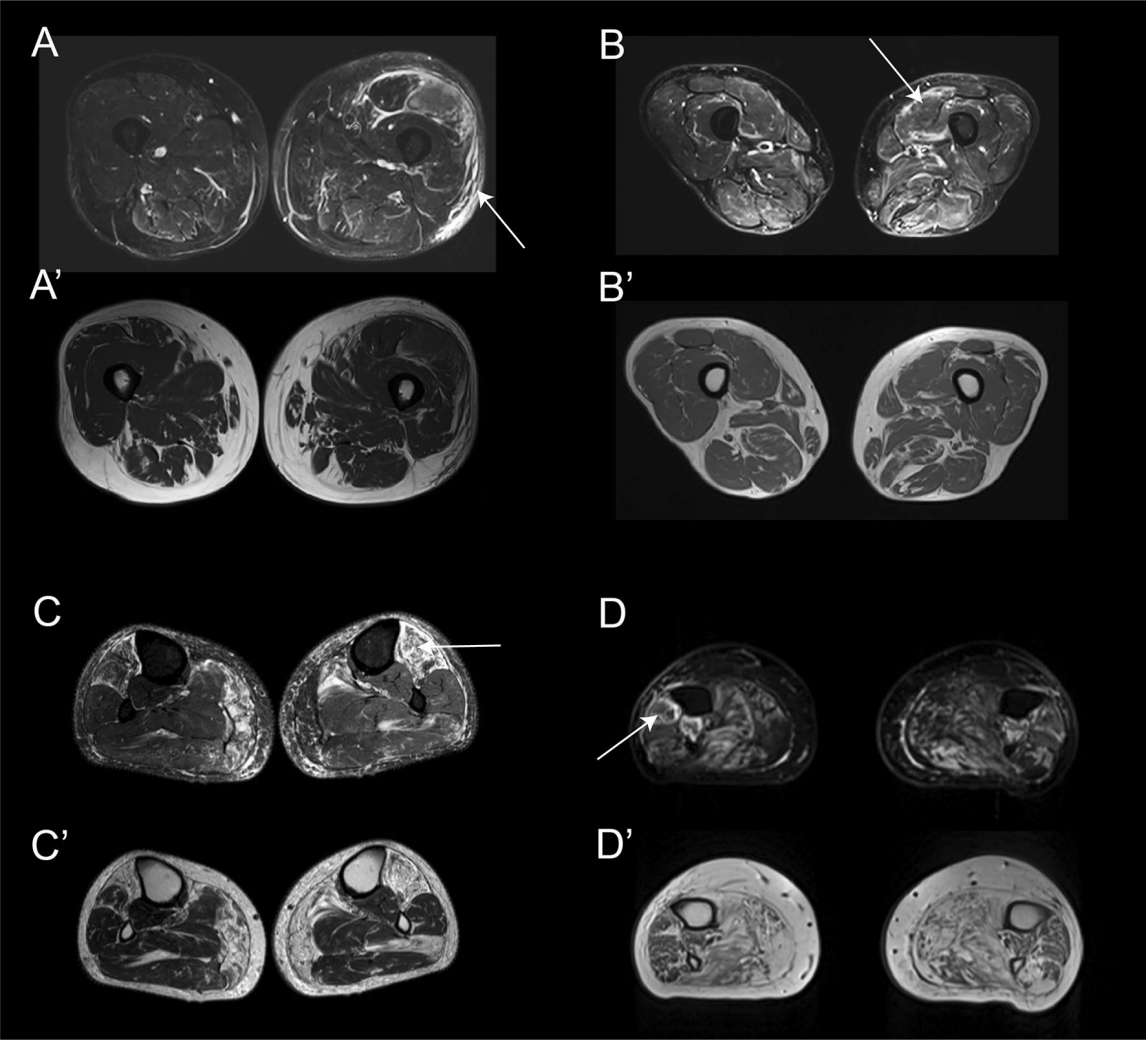
Examples of STIR enhancement in patients with mutations in the VCP gene. The figure shows different examples of STIR enhancement in patients with mutations in the VCP gene. **A** Enhancement of the subcutaneous tissue (arrow), perifascicular (double arrow) and vastus lateralis muscle (asterisk). **A’** T1w image of the same slice showing. No fat replacement in the area enhanced in STIR. **B** Enhancement of the periphery of the vastus lateralis muscle in STIR while B’ shows no fat replacement in that muscle. **C** Enhacement of the whole volume of tibialis anterior which is already replaced by fat in T1 as shown in **C’**. **D** Enhancement of periphery of peroneus muscle (arrow) which is partially replaced by fat as shown in **D’**

STIR imaging was available for 66 patients. Of those, 50 (75.7%) had an increased signal intensity in at least one muscle affecting the lower leg only (36 patients, 54.5%) or in association with the thigh (11/66, 16.6%). The muscles with more frequent STIR abnormalities were the *tibialis anterior* (38 patients, 57.7%) and the *soleus* (35 patients, 53.0%) (Supplemental Fig. 3). A distal to proximal gradient of STIR hyperintensity was observed in 27 patients (40.9%). STIR hyperintensity was observed affecting the skeletal muscles, but also the surrounding fascia, intermuscular tissue and the subcutaneous tissue as shown in Fig. 6.

### MRI rules for the diagnosis of VCP-MSP disease myopathy

**Fig. 7:**
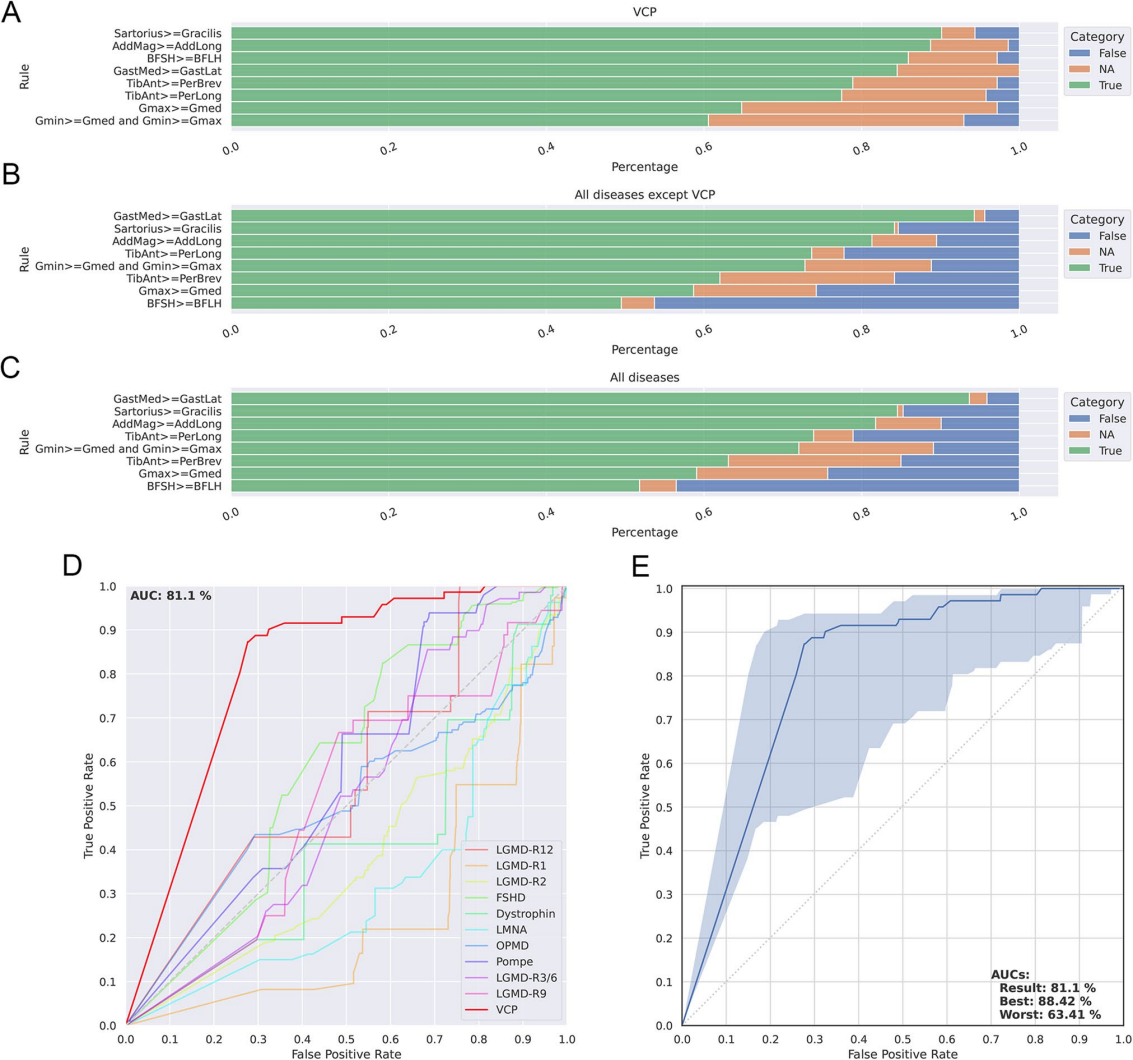
Validation of diagnostic rules identified in VCP myopathy. Figure shows the metrics obtained to validate the rules proposed for VCP myopathy. **A** Results of the analysis of each rule applied to the cohort of VCP patients. **B** Results of the analysis of each rule applied to a cohort of 978 MRIs of 10 different neuromuscular diseases. **C** Results of the analysis of each rule applied to a cohort of VCP patients and 978 MRIs of 10 different neuromuscular diseases. Green, orange and blue in **A**, **B** and **C** corresponds to percentage of patients meeting the rule, percentage of patients with missing data and percentage of patients not meeting the rules, respectively. **D** Comparison of ROC curves obtained after applying the rules generated to MRIs of patients with different neuromuscular diseases. **E** ROC curve obtained after applying the rules generated to the cohort of patients with VCP myopathy. The blue area shows an interval of confidence including of missing variables where the upper limit shows the ROC curve if all missing variables were meeting the rules and the lower limit shows the ROC curve if all missing variables were not meeting the rules. AUC: Area under the curve

Based on the patterns observed we identified a series of potential diagnostic rules and applied them to the present cohort of *VCP*-MSP disease patients. Table 1 shows the accuracy of each individual rule to predict the diagnosis of *VCP*-MSP disease. As described in the methods section, we created an algorithm taking into account all rules defined and applied it to a large cohort of MRI scans including 70 *VCP*-MSP disease patients and 1089 MRI scans of patients included in the Myo-Guide study [35]. The AUC of the ROC curve was 81.1 when predicting the diagnosis of *VCP*-MSP disease, which was higher than the other diagnoses present in the Myo-Guide study (Fig. 7).

**Table 1:** MRI rules described for the diagnosis of VCP myopathy

Rule	Accuracy (%)
Fat pockets	95.7
Serratus anterior, trapezius and latissimus dorsii more or equally affected than infraspinatus, supraspinatus and subscapularis	100
At least one abdominal and paraspinal muscles affected	100
Gluteus minor more or equally affected than gluteus medius	58.5
Gluteus medius more or equally affected than gluteus maximus	85.7
Sartorius more or equally affected than gracilis	90
Adductor magnus more or equally affected than adductor longus	98.3
Vasti muscles more or equally affected than the rectus femoris	68.8
Biceps short head more or equally affected than the biceps long head	90.1
Gastrocnemius medialis more or equally affected than the gastrocnemius lateralis	92.5
Tibialis anterior more or equally affected than the peronei muscles	89.5

### Identification of signs of Paget’s disease of bone on the MRI

**Fig. 8:**
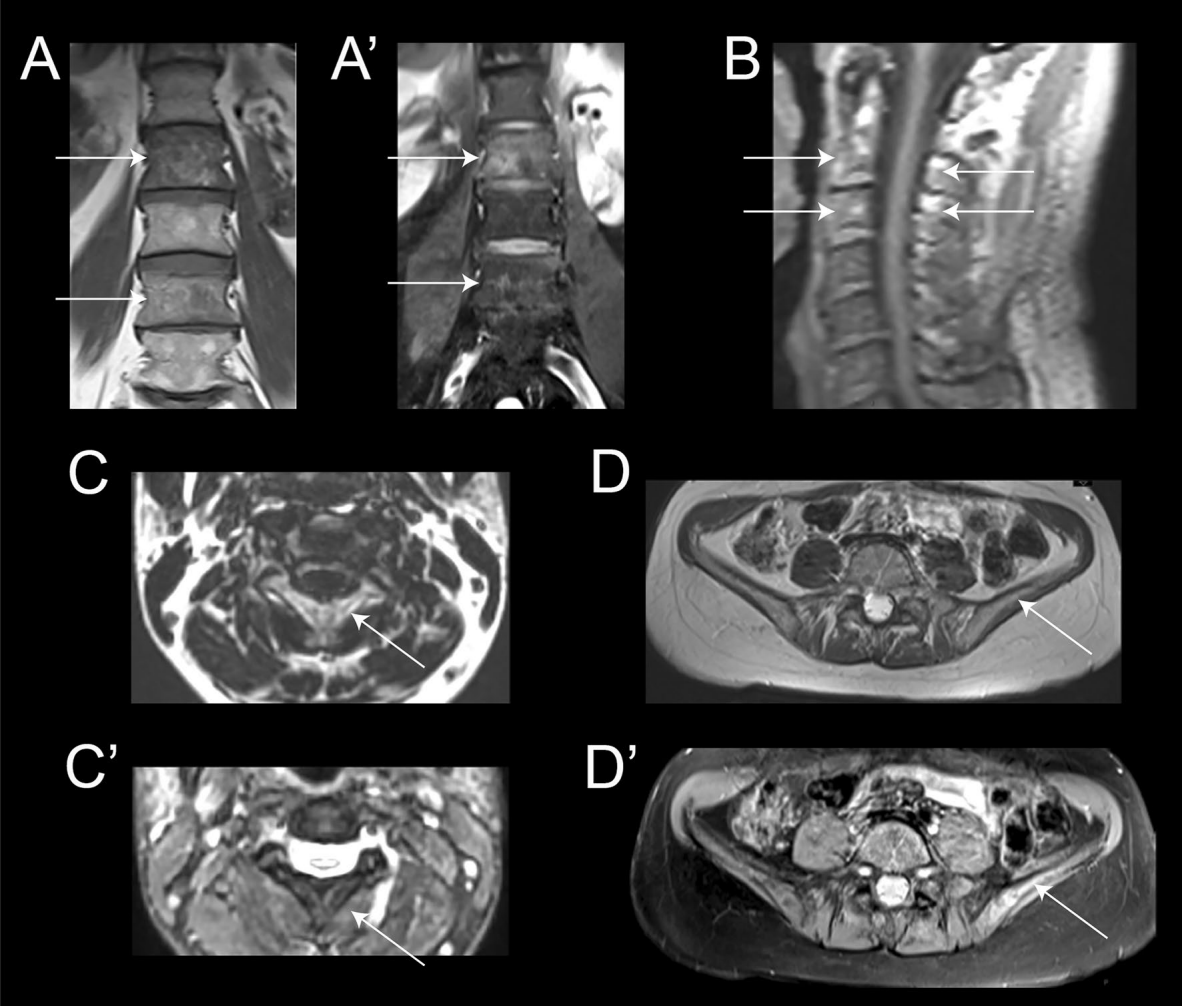
Examples of MRI finding in patients with Paget disease of the bone included in this study. Coronal views T1 (**A**) and T2 with fat saturation (**A’**)-weighted images of the lumbar spine showing heterogeneity of the signal of bone marrow at L2 and L4 levels compared to L1 and L3 (arrows). Coarsed trabeculae are more visible on T1 and edema of the vertebral body on T2 fat sat. Sagital (**B**) and axial (**C** and **C’**) T1-weighted and axial T2 with fat saturation views centered on the upper cervical spine. Signal heterogeneity, cortical thickening and bone enlargement of the entirety of C2 and C3 vertebrae is detectable (arrows). Axial T1 (**D**) and T2 (**D’**) with fat saturation-weighted images centered on the iliac crest. Bone changes are very subtle on T1 but bone edema and heterogeneity is more visible on T2 on the left side (arrow) especially in comparison with the right side

A total of 12 patients out of the 71 included were already diagnosed with PDB. We identified changes compatible with PDB in eight of them. In six cases, the changes were only visible in the pelvic area and especially in the iliac bone, while in one case the changes were visible in the lumbar vertebrae and in another case in the cervical vertebrae C2-C3, characterized by coarsened trabeculae, cortical thickening and bone enlargement of the body of the two vertebrae (Fig. 8).

We were not able to detect any bone changes in the remaining 4 cases, although in one case the examination was restricted to the lower limbs without pelvic coverage, and in another case, the whole-body examination was performed with a large gap between slices.

### Influence of demographic, clinical and genetic features on muscle involvement

We did not identify differences in the degree of fat replacement in any muscle based on the gender of the patients. Neither the genotype nor the phenotype at the time of MRI correlated with the pattern or severity of the fat replacement. We did not identify differences in the pattern of muscle involvement between patients with an exclusive myopathic presentation and patients with associated motor neuron involvement. We observed a significant positive correlation between age of the patients when MRI was performed and the total amount of fat identified in the muscles, although the correlation coefficient was low (*p = *0.01, *R = *0.33). There was a statistical trend toward a positive significant correlation between the degree of fat replacement and functional status (*p = *0.06, *R*: 0.35). We did not observe any correlation between MRI score and respiratory function using FVC percentage predicted, and there were not differences in MRI score between ventilated and non-ventilated patients.

### Influence of disease progression on skeletal muscle involvement

To investigate the sequence of muscle involvement as the disease progresses, we classified the patients into 3 groups depending on their functional status (ambulant, requiring aids for walking and wheelchair users) and calculated the median value of fat replacement of muscle tissue of the lower limbs. The heatmaps obtained suggested a pattern of disease progression. The most affected muscles in the earliest stages of the disease were the *gluteus minimus*, the *sartorius* and the *adductor magnus*. In the intermediate stage, besides the aforementioned muscles, we observed common involvement of the short head of the *biceps femoris*, the *gastrocnemius medialis*, *soleus*, *tibialis anterior* and *extensor digitorum* muscles in the legs. Finally, in the advanced cases requiring a wheelchair, pelvic and proximal muscles of the thigh became severely affected including the *vasti* and the *semimembranosus* muscles (Supplemental Fig. 4).

## Discussion

Mutations in the *VCP* gene produce in most of the patients a myopathy that can be associated with other phenotypes such as dementia, PDB, motor neuron disease or polyneuropathy [20]. The diagnosis of the disease can sometimes be difficult as patients can present with a huge variety of neurological symptoms, weakness being the most common one [21]. However, weak muscles also vary considerably from one patient to another, especially at early stages, which translates into a plethora of potential combinations of muscles involved. Muscle MRI is useful in the diagnostic process of many neuromuscular diseases [23]. Here, we reviewed the muscle MRI of 71 patients included in the “VCP International Study,” representing to our knowledge the largest cohort of *VCP*-MSP disease patients studied by muscle MRI so far. Our analysis has allowed us to identify some characteristic features that, although not pathognomonic, could be useful for the diagnosis. The most common feature observed by MRI was the presence of “fat pockets” also described as “popcorn appearance” in the skeletal muscles. This finding although suggestive, is not specific for *VCP*-MSP disease patients as it has also been described in neurogenic diseases such as spinal muscular atrophy (SMA), distal motor neuropathies and Charcot Marie Tooth disease but also in other myopathies such as neutral lipid storage disease [19, 36]. The existence of “fat pockets” in MRI scans of *VCP*-MSP disease patients may have different origins. It could suggest a component of neurogenic involvement ranging from ALS or lower motor neuron involvement to mild involvement in patients with combined myopathic and neuropathic features. Supporting this hypothesis, muscle biopsies of *VCP*-MSP disease patients can also show a combination of myopathic and neurogenic changes [29]. Interestingly, we have not observed differences in the pattern of muscle involvement between patients with a predominant myopathic or a neurogenic phenotype, suggesting that there is a continuum between the two phenotypes, and the underlying pathology producing the disease is similar. But the presence of fat pockets that in many patients adopt a lineal distribution as seen in the coronal slices suggest that groups of muscle fibers closely located together degenerate at the same time and are replaced by fat, which suggests a kind of programmed death spreading from one fiber to the other or a grouped muscle fiber necrosis.

Muscle replacement by fat is a consistent finding in the disease, affecting mainly scapular, trunk, pelvic and thigh muscles. The combination of muscles affected in the periscapular region including the *trapezius*, *latissimus dorsii* and the *serratus anterior* with sparing of the *subscapularis* and the *infra/supraspinatus* is very similar to the pattern described in FSHD, which is one of the potential clinical differential diagnosis of *VCP*-MSP disease myopathy [37]. We have identified other radiological features shared with FSHD, as the high frequency of asymmetries, a common involvement of paraspinal and abdominal muscles, hyperintensities in STIR imaging in several muscles and the common involvement of the *tibialis anterior, gastrocnemius medialis* and *soleus* in the legs [38]. The involvement of the thigh is also similar but there are some differences between these two diseases. In *VCP*-MSP disease, there is a predominant involvement of the *adductor magnus*, the *vasti*, the *biceps femoris* short head and the *sartorius* muscles with sparing of *rectus femoris* and *adductor longus* muscles. Moreover, the posterior muscles of the thigh tend to be less involved than the *vasti* muscles. On the contrary, in FSHD there is a predominant involvement of the posterior muscles of the thigh, the *rectus femoris*, *adductor longus* and the *adductor magnus* [38]. *Vasti* muscles, although often involved, tend to be less affected than the posterior muscles of the thigh in FSHD. However, the most important difference between FSHD and *VCP*-MSP disease is the presence of “fat pockets” observed in the *VCP*-MSP disease patients, which per se helps to distinguish *VCP*-MSP disease from many other myopathies, including LGMD which are another of the differential diagnosis of *VCP*-MSP disease myopathy. In this regard, the fact that most *VCP*-MSP disease patients have sparing of the *subscapularis* and the *infra/supraspinatus*, early involvement of abdominal muscles and involvement of the *sartorius* are clues that can be helpful to differentiate from LGMD produced by mutations in the *CAPN3*, *FKRP* or the sarcoglycan genes which are diseases that can clinically mimic *VCP*-MSP disease, especially if patients present with limb girdle muscle weakness associated with scapular winging [39–41]. Another important differential diagnosis of VCP-MSP is acquired IBM. The MRI of IBM has been broadly described and include distinctive features compared to VCP including fat replacement of the flexor fingers in the forearm, a gradient of fat replacement in the quadriceps from distal to proximal and hyperintensity in the signal on STIR in several muscles [42]. These features could be useful to distinguish IBM from VCP-MSP.

We have described a series of diagnostic rules based on the pattern of MRI involvement that could be helpful to distinguish *VCP*-MSP disease from other diseases and implemented a new algorithm to validate these rules in a large cohort of patients with muscular disorders including several LGMDs, DMD/BMD and FSHD [35]. Our results show that there is not a single characteristic pathognomonic for *VCP*-MSP disease patients, but the combination of all rules can predict the diagnosis with a high accuracy, reinforcing the utility of muscle MRI in daily clinics, especially if new variants are found in the *VCP* gene.

Another striking finding on the MRI is the presence of hyperintensities in STIR which reflect the presence of free water in the muscle and have been associated with inflammation, muscle necrosis or denervation [22]. Muscle biopsies of *VCP*-MSP disease patients can present scattered inflammatory cells along with denervation, which are two common causes of increase in the STIR [26, 43]. However, the existence of subcutaneous hyperintensities is mainly observed in patients with inflammatory myopathies who have a component of fasciitis, which has not been described so far in *VCP*-MSP disease patients to our knowledge [44, 45]. These results suggest that there could be an inflammatory process or edema affecting the fascia in some cases, although a biopsy of this region is needed to determine the reason for this enhancement. Moreover, these results suggest that the hyperintensities observed could be a sign of disease activity as has been shown in many other neuromuscular conditions [38].

One of the advantages of assessing patients with MRI is that it can also be helpful to identify bone lesions that could suggest the diagnosis of PDB and help in the diagnosis of patients. However, it is important to consider that the sensitivity of MRI is lower compared to X-ray or CT scan for the diagnosis of bone lesions [31]. Here, we have observed changes in eight of the 12 patients known to have PDB, while none of the remaining 58 patients had any suggestive change. Most of the changes were observed in the pelvis of the patients and are similar to previous descriptions of patients with PDB not associated with mutations in *VCP*. Moreover whole-body MRI can also allow to study the brain, which is relevant in this disease. In most of the centers, a neck coil is used to study brain, face and neck muscle, and therefore, it is possible to plan an extended study of the brain using sequences such as T1 or T2. Here we observed parieto-occipital atrophy in one of the patients scanned.

We have also analyzed how the disease progresses in terms of muscle involvement from early stages, in which patients are still ambulant, to advanced stages, when patients have lost ambulation and are non-ambulant. This analysis raises interesting questions about factors affecting the rate of muscle degeneration. Despite the variability of the phenotype from one patient to the other, we have observed that there are certain muscles affected early in the progression of the disease such as *gluteus minor* or *adductor magnus* while others, such as *gluteus medius* or *maximus* are only affected in advanced cases. In addition, while a group of muscles could become involved at the same time, the progression of fat replacement between muscles in a group could also vary. For example, in most patients, the *gluteus minimus* becomes completely replaced by fat at early stages of disease progression, while the *adductor magnus*, while also involved in ambulant patients, is often not completely replaced in advanced cases. It is tempting to hypothesize that muscles with a slower progression could express proteins that protect them from rapid degeneration. Despite previous attempts to investigate this in many neuromuscular disorders, it is still not clear why muscle degeneration shows a different rate of progression [46, 47]. The clear understanding of the pattern of severely involved and spared muscles demonstrated here should allow for more focused investigations in the future.

This study has several limitations that need to be mentioned. First, MRIs were performed in different scanners and using different protocols of acquisition, which has complicated the interpretation of some cases forcing us to not include nine scans. However, due to the popularization of MRI for diagnosis across neuromuscular units, T1w and STIR are today considered standard sequences and many specialized centers have implemented the standardized protocols published in guidelines [48]. Due to the retrospective nature of the project, as we collected the MRIs already performed for diagnosis purposes, there were only a few whole-body MRIs limiting the information obtained from the trunk, upper limbs and cranial muscles. However, the data obtained have identified a pattern of involvement in these regions, suggesting a combination of muscles affected or spared that can be useful for the diagnosis. Third, we did not obtain muscle function tests of the patients when the MRI was performed, and therefore, the correlations made here were only done with the ambulatory status limiting the validation of MRI as an outcome measure that correlates with muscle function in *VCP*-MSP disease myopathy.

In summary, the present study, performed in the framework of the “VCP International Study,” allowed the collection of a large number of MRIs of *VCP*-MSP disease patients and the study of the main MRI features, which can be useful to guide the genetic diagnosis of patients. Fat replacement is a constant feature as all symptomatic patients displayed at least one muscle replaced partially or completely by fat. The most striking MRI characteristic is the presence of patches of fat progressing toward a confluent accumulation of fat surrounding areas of normal muscle, known as “fat pockets,” that were observed in more than 90% of the patients studied. Fat replacement was associated with STIR hyperintensities in many patients that involved not only skeletal muscles but also the fascia and subcutaneous tissue surrounding the muscle. Although there was a wide variability in muscles that were affected, we identified a series of diagnostic rules based on the pattern of fat replacement that can help distinguish *VCP*-MSP disease from other neuromuscular disorders.

### Supplementary Information

Below is the link to the electronic supplementary material.

Supplementary file1 Supplemental Figure 1: Linear distribution of fat replacement in the skeletal muscles of patients with mutations in the VCP gene. The figure shows examples of the linear replacement of fat replacement in skeletal muscles of the pelvis (A and B), thigh (C) and lower leg (D). (TIF 1587 KB)

Supplementary file2 Supplemental figure 2: Examples of paraspinal involvement of patients with mutations in the VCP gene. T1 weighted axial images of two VCP patients, at cervical, thoracic and lumbar levels. There is a fatty infiltration of the epi-axial spinal muscles(rotatores, multifidus, longissimus and ilio-costalis) more pronounced than in hypo-axial muscles(psoas and quadratus lomborum), with a cranio-caudal and mediolateral gradient of involvement (better preservation of the rotatores) as shown in cervical (A and B), thoracic (C and D) and lumbar (E and F) levels. (TIF 227 KB)

Supplementary file3 Supplemental figure 3: Heatmap showing pattern of enhancement on STIR sequence. Heatmap showing enhancement on STIR sequence of the muscles of the pelvis, thigh and leg. Patients and muscles are ordered according to hierarchical clustering. The score of a muscle in a patient is indicated by the color of the square. Column of the left displays the phenotype of patient divided in isolated myopathy (blue) or lower motor neuron involvement associated or not to myopathy (green). (TIF 740 KB)

Supplementary file4 Supplemental figure 4: Heatmap showing the progression of the muscle involvement related to the ambulatory status of patients. Patients were divided into three groups depending on their ambulatory status for the analysis of the progression of muscle involvement. Muscles (columns) are ordered according to hierarchical clustering with increasing grading of muscle fatty transformation in T1-W imaging from the right to the left. The score of a muscle per group is indicated by the colour of the square. We obtained a pattern of the progression of the disease in muscles of the pelvis, thighs and lower legs related to the ambulatory status. (TIF 842 KB)

Supplementary file5 (DOCX 19 KB)

Supplementary file6 (DOCX 14 KB)

## Data Availability

Data obtained are available for further analysis by contacting the corresponding author of the paper.
