# Correction to: Analysis of muscle magnetic resonance imaging of a large cohort of patient with VCP‑mediated disease reveals characteristic features useful for diagnosis

**DOI:** 10.1007/s00415-023-12178-z

**Published:** 2024-02-13

**Authors:** Diana Esteller, Marianela Schiava, José Verdú-Díaz, Rocío-Nur Villar-Quiles, Boris Dibowski, Nadia Venturelli, Pascal Laforet, Jorge Alonso-Pérez, Montse Olive, Cristina Domínguez-González, Carmen Paradas, Beatriz Vélez, Anna Kostera-Pruszczyk, Biruta Kierdaszuk, Carmelo Rodolico, Kristl Claeys, Endre Pál, Edoardo Malfatti, Sarah Souvannanorath, Alicia Alonso-Jiménez, Willem de Ridder, Eline De Smet, George Papadimas, Constantinos Papadopoulos, Sofia Xirou, Sushan Luo, Nuria Muelas, Juan J. Vilchez, Alba Ramos-Fransi, Mauro Monforte, Giorgio Tasca, Bjarne Udd, Johanna Palmio, Srtuhi Sri, Sabine Krause, Benedikt Schoser, Roberto Fernández-Torrón, Adolfo López de Munain, Elena Pegoraro, Maria Elena Farrugia, Mathias Vorgerd, Georgious Manousakis, Jean Baptiste Chanson, Aleksandra Nadaj-Pakleza, Hakan Cetin, Umesh Badrising, Jodi Warman-Chardon, Jorge Bevilacqua, Nicholas Earle, Mario Campero, Jorge Díaz, Chiseko Ikenaga, Thomas E. Lloyd, Ichizo Nishino, Yukako Nishimori, Yoshihiko Saito, Yasushi Oya, Yoshiaki Takahashi, Atsuko Nishikawa, Ryo Sasaki, Chiara Marini-Bettolo, Michela Guglieri, Volker Straub, Tanya Stojkovic, Robert Y. Carlier, Jordi Díaz-Manera

**Affiliations:** 1 Neurology Department, Hospital Clinic de Barcelona, Universitat de Barcelona, Barcelona, Spain; 2 John Walton Muscular Dystrophy Research Centre, Newcastle University Translational and Clinical Research Institute and Newcastle Hospitals NHS Foundation Trust, Center for Life, Central Parkway, Newcastle Upon Tyne, NE13BZ United Kingdom; 3 APHP, Centre de Référence des Maladies Neuromusculaires, Institut de Myologie, Centre de Recherche en Myologie, Sorbonne Université, APHP, Hôpital Pitié-Salpêtrière, Paris, France; 4 Department of Radiology, Assistance Publique-Hôpitaux de Paris (AP-HP), DMU Start Imaging, Raymond Poincaré Teaching Hospital, Garches, France; 5 Département de Neurologie Hôpital Raymond-Poincaré Garches France Inserm U1179, Garches, France; 6 Servicio de Neurología. Hospital Virgen de la Candelaria, Tenerife, Spain; 7 Neuromuscular Diseases Unit, Neurology Department, Institut d’Investigació Biomèdica Sant Pau (IIB SANT PAU), Hospital de la Santa Creu i Sant Pau, Barcelona, Spain; 8 Center for Biomedical Network Research on Rare Diseases (CIBERER), Instituto de Salud Carlos III, Madrid, Spain; 9 Unidad de Enfermedades Neuromusculares, Servicio de Neurología, Instituto de Investigación imas12, Hospital 12 de Octubre, Madrid, Spain; 10 Unidad de Enfermedades Neuromusculares, Servicio de Neurología, Hospital Virgen del Rocio, Seville, Spain; 11 Centro de Investigación Biomédica en Red en Enfermedades Neurodegenerativas (CIBERNED), Barcelona, Spain; 12 Department of Neurology, Medical University of Warsaw, ERN EURO NMD, Warsaw, Poland; 13 UOC di Neurologia e Malattie Neuromuscolari, AOU Policlinico “G. Martino”, Rome, Italy; 14 Neurologie, Neuromusculair Referentiecentrum, Universitaire Ziekenhuizen, Leuven, Belgium; 15 Neurology Department, University of Pécs, Pécs, Hungary; 16 Université Paris Est, U955 INSERM, Centre de Référence de Pathologie Neuromusculaire Nord-Est-Ile-de-France, Henri Mondor Hospital, EURO-NMD, 94010 Creteil, France; 17 Neurology Department, Universitary Hospital Antwerpen, Edegem, Belgium; 18 Department of Neurology, Eginition Hospital, Medical School, NKUA, ERN, EURO NMD, Athens, Greece; 19 Neurology Department, Huashan Hospital, Fudan University, Shangai, China; 20 Neuromuscular Diseases Unit, Neurology Department, Hospital Universitari I Politècnic La Fe, Valencia, Spain; 21 Neuromuscular Reference Centre, ERN-EURO-NMD, Warsaw, Poland; 22 Neuromuscular and Ataxias Research Group, Instituto de Investigación Sanitaria La Fe, Valencia, Spain; 23 Department of Medicine, Universitat de València, Valencia, Spain; 24 Unitat de Malalties Neuromusculars, Servei de Neurologia, Hospital Germans Tries I Pujol, Badalona, Spain; 25 UOC di Neurologia, Fondazione Policlinico Universitario A. Gemelli IRCCS, Rome, Italy; 26 Tampere Neuromuscular Center, Tampere University Hospital, Tampere, Finland; 27 Folkhalsan Genetic Institute, Helsinki University, Helsinki, Finland; 28 Sree Chitra Tirunal Insitute for Medical Sciences and Technology, Thiruvananthapuram, India; 29 Department of Neurology, Friedrich-Baur-Institute, LMU Clinics, Munich, Germany; 30 Neurology Department, Biodonostia Health Research Institute, Donostia, Spain; 31 Department of Neurosciences, University of Padova, Padua, Italy; 32 Department of Neurology, Institute of Neurological Sciences, Queen Elizabeth University Hospital, Glasgow, Scotland, UK; 33 Heimer Institut for Muscle Research, Klinikum Bergmannsheil Ruhr, University Bochum, Bochum, Germany; 34 University of Minnesota, Minneapolis, USA; 35 Centre de Référence des Maladies Neuromusculaires Nord/Est/Ile-de-France and ERN-EURO-NMD, Neurology Department, Hôpitaux Universitaires de Strasbourg, Strasbourg, France; 36 Neurology Department, Medical University of Vienna, Vienna, Austria; 37 Leiden University Medical Center, Leiden, The Netherlands; 38 Department of Medicine (Neurology), The Ottawa Hospital, Ottawa, Canada; 39 Departamento de Neurología y Neurocirugía, Hospital Clínico Universidad de Chile, Santiago de Chile, Chile; 40 Department of Neurology, Johns Hopkins University School of Medicine, Baltimore, USA; 41 Department of Neuromuscular Research, National Institute of Neuroscience, National Center of Neurology, Tokyo, Japan; 42 Department of Neurology, National Center Hospital, NCNP, Tokyo, Japan; 43 Department of Neurology, Kagawa Prefectural Central Hospital, Kagawa, Japan; 44 Department of Neurology, Kinki Central Hospital, Hyogo, Japan; 45 Department of Neurology, Graduate School of Medicine, Dentistry and Pharmaceutical Sciences, Okayama University, Okayama, Japan

**Correction to: Journal of Neurology (2023) 270:5849–5865** 10.1007/s00415-023-11862-4.

In the original version of this article, third author name and affiliation were missing and should have been

José Verdú-Díaz

John Walton Muscular Dystrophy Research Centre, Newcastle University Translational and Clinical Research Institute and Newcastle Hospitals NHS Foundation Trust, Center for Life, Central Parkway, Newcastle Upon Tyne, NE13BZ, United Kingdom.

And author name Benedikt Schoser was incorrectly written as Benedikt Schöser.

The original article has been corrected.

