# Supplementary figures and images for: Analysis of muscle magnetic resonance imaging of a large cohort of patient with VCP-mediated disease reveals characteristic features useful for diagnosis
Source: J Neurol. 2023 Aug 21;270(12):5849–65. doi: 10.1007/s00415-023-11862-4 (PMC10632218; doi:10.1007/s00415-023-11862-4)

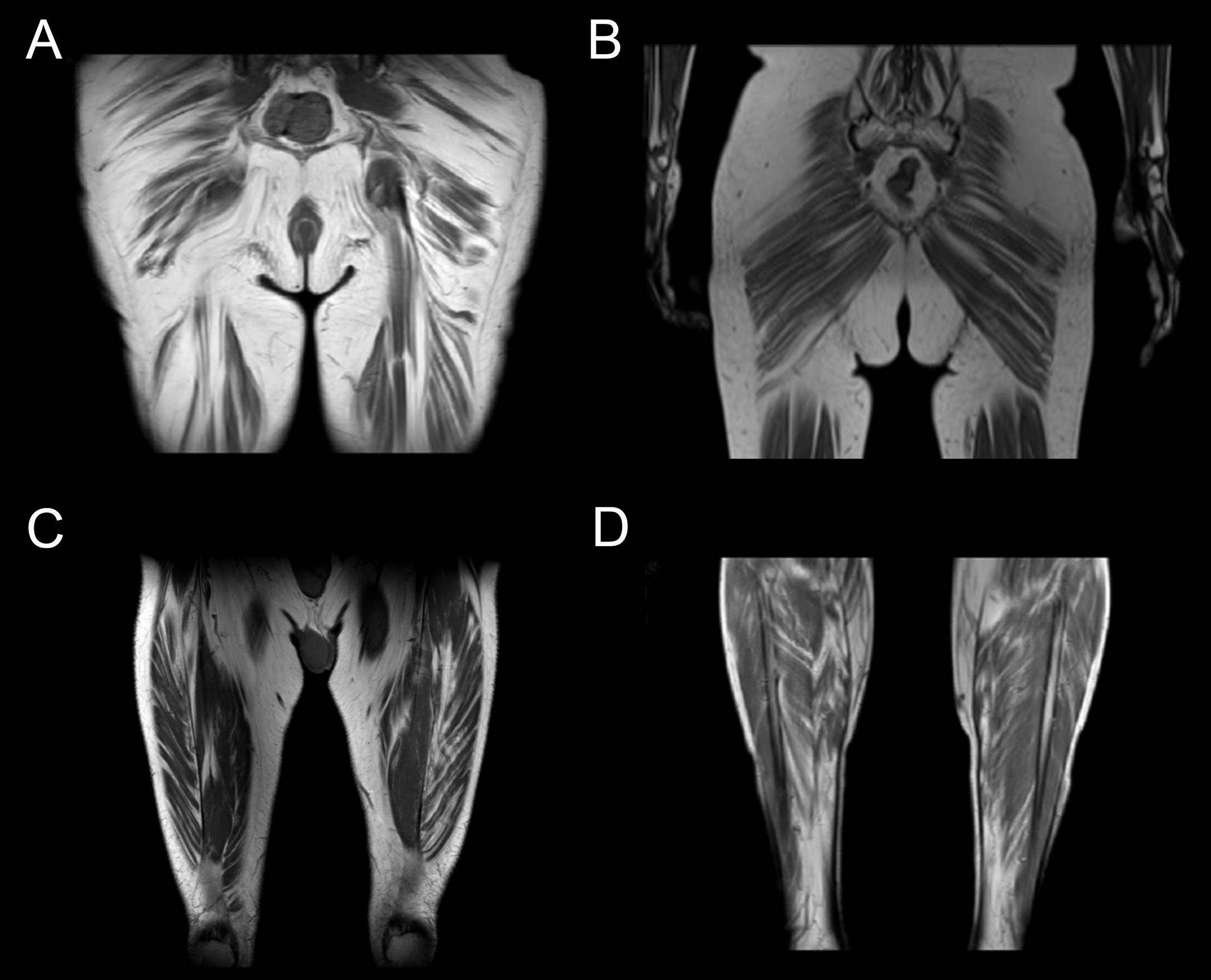

Supplement: Supplementary file 1 — Supplementary file1 Supplemental Figure 1: Linear distribution of fat replacement in the skeletal muscles of patients with mutations in the VCP gene. The figure shows examples of the linear replacement of fat replacement in skeletal muscles of the pelvis (A and B), thigh (C) and lower leg (D). (TIF 1587 KB) [file 415_2023_11862_MOESM1_ESM.tif]

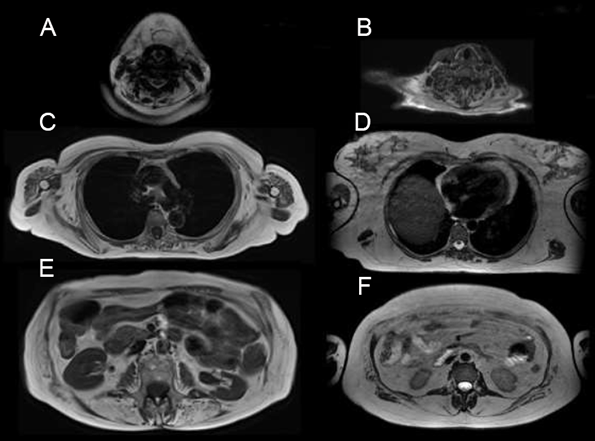

Supplement: Supplementary file 2 — Supplementary file2 Supplemental figure 2: Examples of paraspinal involvement of patients with mutations in the VCP gene. T1 weighted axial images of two VCP patients, at cervical, thoracic and lumbar levels. There is a fatty infiltration of the epi-axial spinal muscles(rotatores, multifidus, longissimus and ilio-costalis) more pronounced than in hypo-axial muscles(psoas and quadratus lomborum), with a cranio-caudal and mediolateral gradient of involvement (better preservation of the rotatores) as shown in cervical (A and B), thoracic (C and D) and lumbar (E and F) levels. (TIF 227 KB) [file 415_2023_11862_MOESM2_ESM.tif]

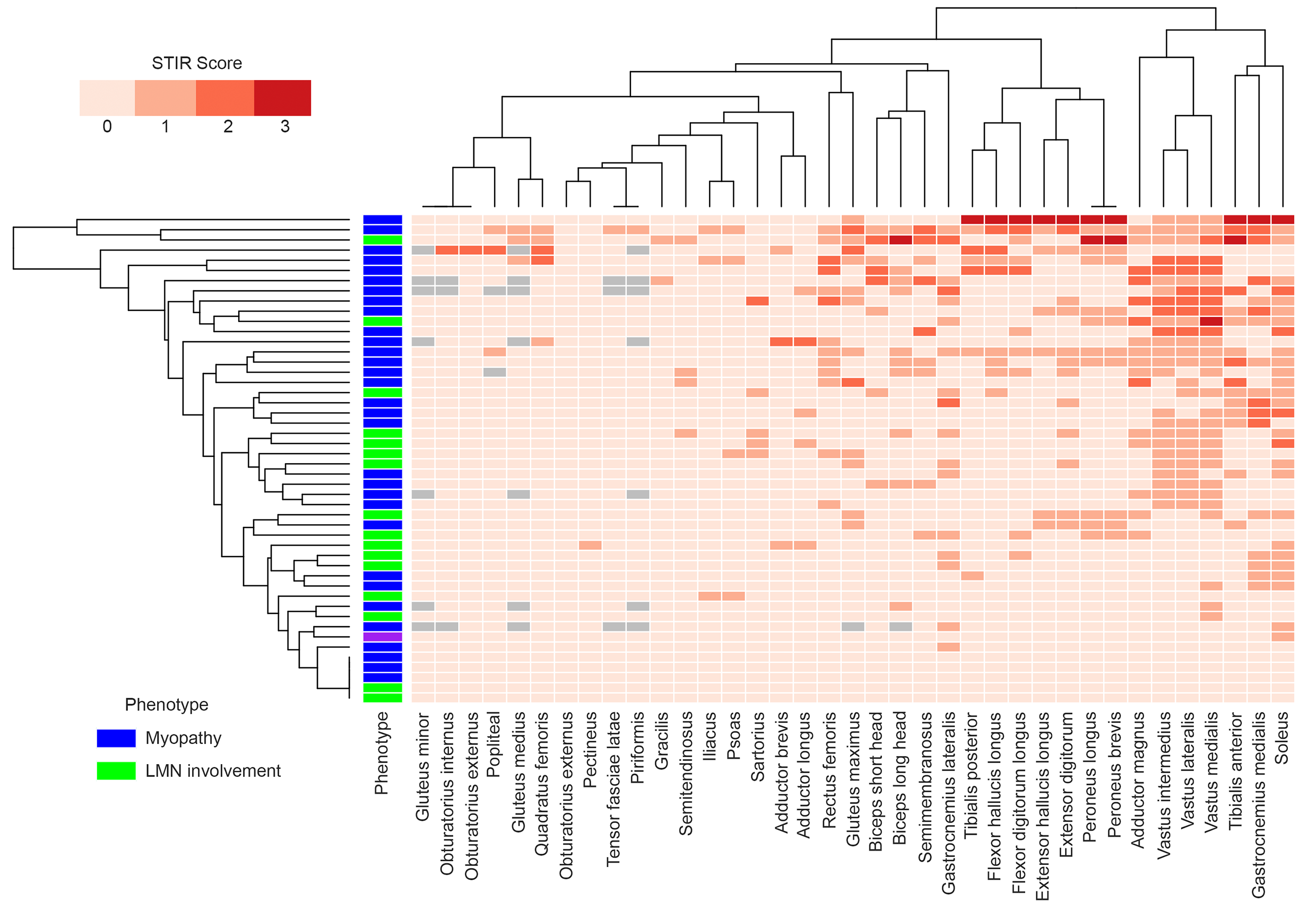

Supplement: Supplementary file 3 — Supplementary file3 Supplemental figure 3: Heatmap showing pattern of enhancement on STIR sequence. Heatmap showing enhancement on STIR sequence of the muscles of the pelvis, thigh and leg. Patients and muscles are ordered according to hierarchical clustering. The score of a muscle in a patient is indicated by the color of the square. Column of the left displays the phenotype of patient divided in isolated myopathy (blue) or lower motor neuron involvement associated or not to myopathy (green). (TIF 740 KB) [file 415_2023_11862_MOESM3_ESM.tif]

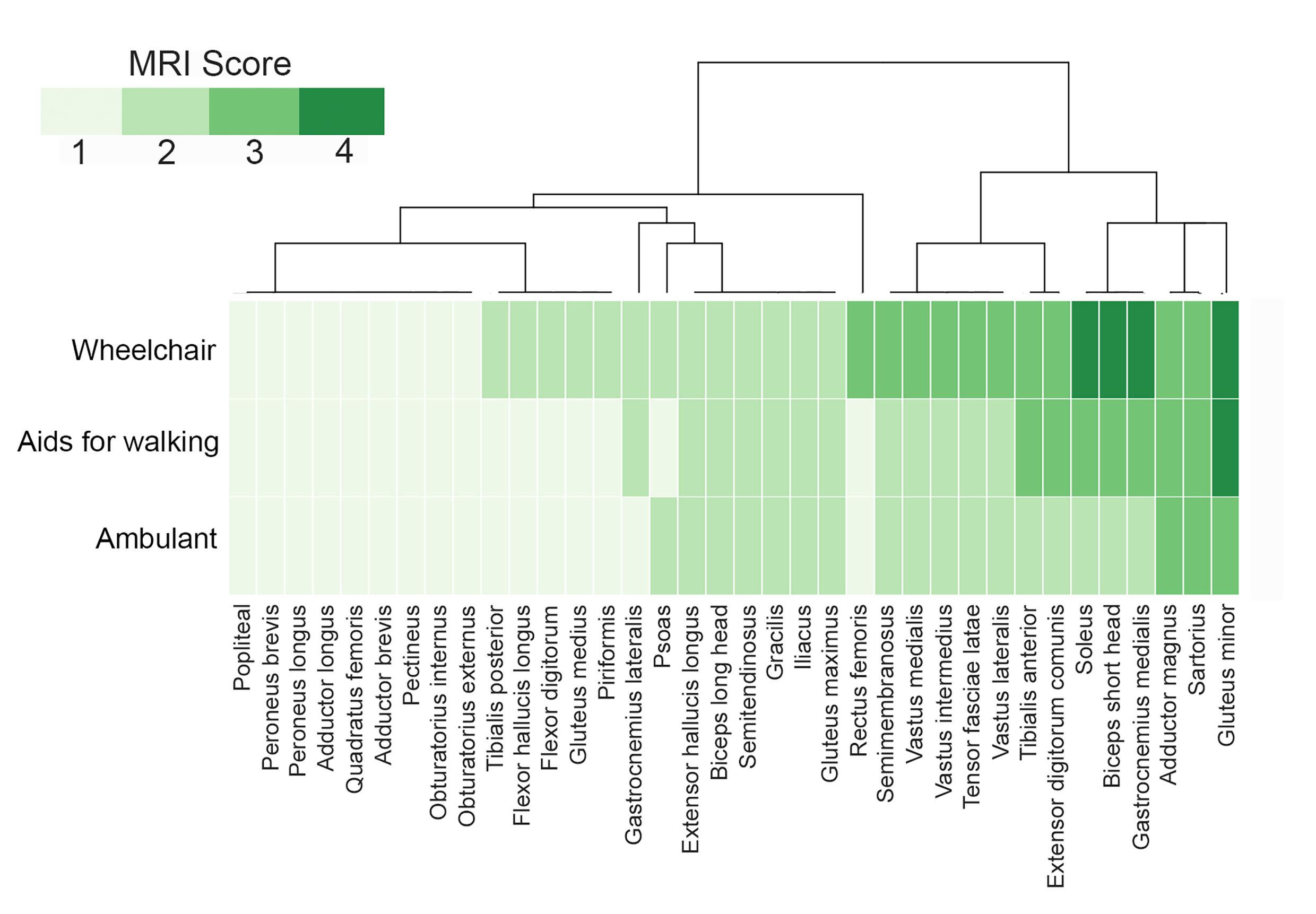

Supplement: Supplementary file 4 — Supplementary file4 Supplemental figure 4: Heatmap showing the progression of the muscle involvement related to the ambulatory status of patients. Patients were divided into three groups depending on their ambulatory status for the analysis of the progression of muscle involvement. Muscles (columns) are ordered according to hierarchical clustering with increasing grading of muscle fatty transformation in T1-W imaging from the right to the left. The score of a muscle per group is indicated by the colour of the square. We obtained a pattern of the progression of the disease in muscles of the pelvis, thighs and lower legs related to the ambulatory status. (TIF 842 KB) [file 415_2023_11862_MOESM4_ESM.tif]
