# Supplementary material for: Analysis of muscle magnetic resonance imaging of a large cohort of patient with VCP-mediated disease reveals characteristic features useful for diagnosis
Source: J Neurol. 2023 Aug 21;270(12):5849–65. doi: 10.1007/s00415-023-11862-4 (PMC10632218; doi:10.1007/s00415-023-11862-4)
Supplement: Supplementary file 5 — Supplementary file5 (DOCX 19 KB) [file 415_2023_11862_MOESM5_ESM.docx]

**Modelling the data to identify rules for diagnosis**

The data on the fatty replacement of different muscles allowed us to identify potential rules for the diagnosis of patients with VCP. We studied the accuracy of these rules in the VCP cohort studied here, but also applied these rules to 1089 muscle MRI of patients with 10 different neuromuscular diseases that were used to build Myo-Guide (28), including Duchenne/Becker muscular dystrophy, limb girdle muscular dystrophy (LGMD) R1 produced by mutations in the *CAPN3* gene, LGMD-R2 produced by mutations in the *DYSF* gene, LGMD-R3 to 6 produced by mutations in the sarcoglycan genes, LGMD-R9 produced by mutations in the FKRP gene, LGMD-R12 produced by mutation in the ANO5 gene, facio-scapulo-humeral muscular dystrophy (FSHD), Pompe disease, oculopharyngeal muscular dystrophy (OPMD) and, patients with muscle disease associated to mutations in the *LMNA* gene. However, we observed that there was a considerable class imbalance in the cohort used for Myo-Guide, with an overrepresentation of some of the diseases, such as FSHD or OPMD, therefore we decided to apply the Matthews Correlation Coefficient (MCC) calculated using the following equation, where TP are tru positive, TN: true negative, FP: false positive and FN: false negative.

$$MCC = \frac{TP \cdot TN - FP \cdot FN}{\sqrt{\left( TP + FP \right) \cdot\left( TP + FN \right) \cdot\left( TN + FP \right) \cdot\left( TN + FN \right)}}$$

MCC is a metric that considers class unbalance and returns a more realistic result ranging between –1 and 1(29). The normalized MCC (Equation X) scales the MCC between 0 and 1.

$$normMCC = \frac{MCC + 1}{2}$$

The Diagnostic Odds Ratio (DOR) measures the performance of a test in detecting and distinguishing positive cases from negative ones. The DOR ranges from 0 to infinity, with values below 1 indicating better performance in detecting negative cases.

$$DOR = \frac{\left( TP + 0.5 \right) \cdot\left( TN + 0.5 \right)}{\left( FP + 0.5 \right) \cdot\left( FN + 0.5 \right)}$$

In the case rules returned a TP, TN, FP or FN of 0, which made the DOR undefined, we added a 0.5 to all values(30).

We developed an algorithm that integrates these rules into a single predictive model. In the algorithm, an array of rules (R) is defined for each patient (p) testing each proposed rule (r). The algorithm is defined as follows: R = [r_0 , r_1 , … r_n ] defined for each patient where r_i are the applicable rules for patient, r_i = 0 if the rule returns False while r_i = 1 if the rule returns True. For each patient we also calculate a weight array W = [w_0 , w_1 , … , w_n ] as $w_{i}=\frac{m_{i}+1}{\sum\left( w_{n} + 1 \right)}$ where m_i is the MCC of rule i and $\sum w_{n}$is the summation of the MCCs of all applicable rules for patient p. The probability of positive diagnosis is then calculated as $P\left( diagnosis \right)=\sum_{i=0}^{n} \left( r_{i} \cdot w_{i} \right)$. A Receiver Operating Characteristic curve (ROC) is used to represent the results of the algorithm applied to our cohort. In order to reduce the impact of the missing data on the results of the algorithm, we applied it but considering the best and worst case scenarios defined as Best Case Scenario (BCS) when all missing data (NAs) in positive class (VCP) are True, while all NAs in negative class are False, and the Worst Case Scenario (WCS) when all NAs in positive class (VCP) are False, while all NAs in negative class are True.
