# Supplementary material for: Analysis of muscle magnetic resonance imaging of a large cohort of patient with VCP-mediated disease reveals characteristic features useful for diagnosis
Source: J Neurol. 2023 Aug 21;270(12):5849–65. doi: 10.1007/s00415-023-11862-4 (PMC10632218; doi:10.1007/s00415-023-11862-4)
Supplement: Supplementary file 6 — Supplementary file6 (DOCX 14 KB) [file 415_2023_11862_MOESM6_ESM.docx]

| **Mutation DNA** | **Mutation 1 prot** | **Frequency (n)** | **Frequency (%)** |
| --- | --- | --- | --- |
| c.464G>A | p.Arg155His | 19 | 27,5% |
| c.277C>T | p.Arg93Cys | 9 | 13,0% |
| c.476G>A | p.Arg159His | 9 | 13,0% |
| c.463C>T | p.Arg155Cys | 7 | 10,1% |
| c.1160A>G | p.Asn387Ser | 3 | 4,3% |
| c.572G>A | p.Arg191Gln | 3 | 4,3% |
| c.572G>C | p.Arg191Pro | 2 | 2,9% |
| c.475C>T | p.Arg159Cys | 2 | 2,9% |
| c.1105A>T | p.Ile369Phe | 1 | 1,4% |
| c.1106T>C | p.Ile369Thr | 1 | 1,4% |
| c.1160A>C | p.Asn387Thr | 1 | 1,4% |
| c.1696-3C>T | p.(?) | 1 | 1,4% |
| c.196G>A | p.Glu66Lys | 1 | 1,4% |
| c.266G>A | p.Arg89Gln | 1 | 1,4% |
| c.283C>T | p.Arg95Cys | 1 | 1,4% |
| c.451A>G | p.Ile151Val | 1 | 1,4% |
| c.463C>A | p.Arg155Ser | 1 | 1,4% |
| c.464G>C | p.Arg155Pro | 1 | 1,4% |
| c.472A>G | p.Met158Val | 1 | 1,4% |
| c.473T>C | p.Met158Thr | 1 | 1,4% |
| c.648A>G | p.Ile216Met | 1 | 1,4% |
| c.785C>G | p.Thr262Ser | 1 | 1,4% |
| c.80T>C | p.Ile27Thr | 1 | 1,4% |
